# New York Odontological Society

**Published:** 1894-05

**Authors:** 

**Affiliations:** New York Odontological Society


### Reports of Society Meetings.

### NEW YORK ODONTOLOGICAL SOCIETY.

A REGULAR meeting of the New York Odontological Society was held on Tuesday evening, February 20, 1894, at the New York Academy of Medicine, No. 17 West Forty-third Street, New York City, Dr. Brockway presiding.

The Secretary, Dr. Starr, read the minutes of the November meeting, which were approved. He also read the minutes of the January meeting, which were likewise approved.

### INCIDENTS OF OFFICE PRACTICE AND CASUAL COMMUNICATIONS.

Dr. George Allan presented an electric mouth-lamp, described under proceedings of Odontological Society of Pennsylvania.

Dr. Wilson then read the following incident of office practice:

A French gentleman, aged about forty, for several years a patient of mine, came to me with the left side of his face, particularly the lower part, swollen very badly—so badly, in fact, that the skin seemed ready to burst. I was greatly surprised, knowing that his teeth on that side above and below were all sound.

An examination—such as could be made, for he could open his jaws but very little—revealed nothing except that about the lower wisdom-tooth the gum was somewhat swollen and inflamed, the tooth itself being only slightly sore to touch. Intuitively, perhaps, I fixed upon that wisdom-tooth as the cause, and advised him that safety from external fistula and eschar of the face, in my opinion, depended on immediate removal of that tooth. Dr. Hasbrouck succeeded in extracting it that same afternoon, how, I can scarcely imagine.

The next day the patient called on me with his face much reduced in size and a very formidable-looking wisdom-tooth in his vest pocket. It had a long bifurcated root extending backward, with a sharp curve upward. Some considerable discharge from the opening followed, but by frequent use of warm extract of witch-hazel and listerine injections with a curved-pointed syringe, the swelling rapidly subsided, and in a few days everything was on the way to recovery.

The interesting feature of the case to me was what should cause such a disturbance after many years of constant use, for he had his full number of teeth above and below, all occluding perfectly and all perfectly sound, the extracted tooth itself having no sign of any decay or disease about it.

*The President.*—Was the tooth thoroughly erupted?

*Dr. Wilson.*—Yes, so that it occluded perfectly with the tooth above it.

*Dr. Payne.*—Last week, the hour was so late when the matter of retaining lower incisors was discussed, that I did not say anything on the subject, but I had something to say about it, and I have written it down.

Under the head of "Casual Communications" at our last meeting, Drs. Davenport and Rhein referred to their method of retaining in place loose lower incisors.

Both methods, if I correctly understand them, held the six lower teeth in a sort of block or rigid condition. I object to any appliance which interferes with the normal movement of teeth. Scientific treatment of natural organs should, as far as possible, conform to natural laws. The movable condition of teeth is an admitted fact. Hence any appliance which holds firmly a movable organ more or less contravenes a natural law. Therefore, I regard rigid appliances less desirable than those which I will describe.

First, I would, if I used the bar at all, form the groove without an undercut, and polish it until it is perfectly smooth. Then accurately fit a gold and platinum bar and anchor it firmly to the cuspids. If the bar is well adjusted and firmly anchored, the teeth will be held in place, and the trouble and annoyance of filling in the loose teeth giving out entirely obviated. We all know how fragile is the end of a lower incisor after an undercut is made sufficient to hold a gold filling around the bar. By leaving the bar loose in the groove greater strength is left to the tooth, and a little movement as in nature is possible. If objection be raised that caries will play an important part, I will say that experience teaches me that such grooves properly polished do not, as a matter of fact, become carious, if at all, in a long time. It will be remembered that at the age such resort is necessary the teeth are dense compared to younger teeth, and besides they are in constant use. I have abandoned the bar over the cutting-edges, but if it is thought desirable to make use of it, I recommend its use in the modified form above indicated.

The method which commends itself as the very best known,—to me at least,—and one that I am tempted to claim to have originated, is to carefully adjust a flattened, highly-polished bar to the posterior surface of the teeth about one-sixteenth to one-quarter of an inch below the top, and to this bar solder fingers of spring gold after the manner of the gold fingers used to hold precious stones. These fingers should be left pointed and straight until the piece is properly adjusted and firmly anchored to the cuspid teeth, then with pliers close the ends of the fingers, not too firmly, but so as to hold the tooth. The gold will show very little indeed in front, as it comes only far enough forward to hold the tooth when the ends are set up with pliers. The piece can be attached to the cuspid teeth with a clasp, or retained in the usual way by cutting a cavity for a gold filling.

An advantage of this method is the absence of the weakening groove and the unsightliness of the bar, or the gold caps referred to by Dr. Davenport; and what is all-important, it does not, as a slight movement is possible, essay to improve on nature. I regard rigid appliances objectionable, and their usefulness for any great length of time questionable. So many bridge-work pieces split on this rock, that at present bridge-workers are adjusting movable pieces, which show that the foregoing law cannot be disregarded without a penalty. I therefore recommend this method as the very best I have ever employed for the purpose.

*Dr. Jackson.*—There are two methods of retaining teeth that have become loose from pyorrhœa or other cause that I practise successfully, to which I would like to call the attention of the Society. The first is a method of grooving the approximal sides of a loose tooth (I am now speaking of a lower incisor), which is done with a thin corundum disk, and rounding the approximal surfaces of the adjoining teeth so that they will fit into the grooves, then springing the loose tooth into position, and retaining it there with a ligature around the adjoining teeth until they remain in contact with it.

This method is especially valuable where there is a tendency for the teeth to become irregular from crowding. One case I have in mind was where the superior incisors had been biting inside the arch. The patient was thirty-eight years of age. After moving the superior incisors in front of the lower ones, it was necessary to support the loose incisor in some way, as it was found that, as the superior incisors pressed against it, there was a tendency for the teeth to crimp. I anticipated this condition, and told the patient I would groove the sides. I did so; the tooth is firmly held, and with a little attention to the gum, I think we shall keep up this healthy condition for some time.

The second method is to use "crib" attachments, and arrange a bar on both the labial and lingual sides of the teeth, the ends of which should be attached by a "crib" attachment to a first bicuspid on each side of the arch.

In Dr. Payne's method, the attachment of springs could extend from a bar on the lingual side of the appliance such as I have described, and do away with the bar on the labial surface. It could be slipped on and off easily and cleansed by the patient, and the anchorage is perfect. The bar in front is a practical method, and can be made in twenty minutes.

*Dr. W. B. Davenport.*—I do not claim any originality for the following few notes of a very interesting case which I had. Its obstinacy, and its being slightly out of the ordinary character of cases, attracted my attention.

The following case of subperiosteal abscess in the roof of the mouth was brought to me for treatment. An examination revealed a fistulous opening on the alveolar ridge where the left superior lateral incisor had been, and a large swelling covering the hard palate on the left side of the median line.

The slightest pressure upon this caused a copious flow of thick, yellow pus from the fistula. The patient was in a weak condition and suffered from insomnia, nervousness, and loss of appetite. I obtained the following history:

About six years before, the pulp having been destroyed, the tooth was filled with gold. The patient remembers that the probing of the root-canal caused acute pain, but the tooth was comparatively comfortable after that for about five years, being sensitive, however, to cold and sweets. Suddenly, soreness of the tooth was noticed, and then a small pustule appeared on the labial face of the gum. Soon there was a discharge of pus from this which continued daily for six months, when the swelling appeared in the roof of the mouth, and the patient, becoming discouraged, had the tooth extracted. Instead of the relief expected, the discharge of pus from the alveolus not only continued, but increased in frequency and amount, and the swelling on the palate became larger. The pain was very intense and involved the entire left side of the face.

A physician was consulted, who lanced the swelling twice, but employed no medication.

The case came under my notice six months after the extraction of the tooth. At that time the abscess was discharging three or four times a day an amount of pus sufficient to saturate a handkerchief. My treatment was as follows:

Seeing the patient daily, I evacuated the abscess by a firm pressure with the finger, upward and forward, and syringed it with a

1 to 5000 solution of bichloride of mercury. Repeating the upward and forward pressure on the roof of the mouth, the solution would return mixed with pus, when I would syringe it again, and again evacuate the sac by the means described, until the solution would return clear. The mouth of the fistula was then plugged with cotton. This was continued daily for two weeks, when an improvement was noticed ; but on relaxing the treatment the abscess returned to its original condition. During the next twenty-three days the treatment was unavoidably irregular, being performed for some time by the patient herself at home, after I had instructed her in the use of the syringe, and also by myself at the office.

The entire time of treatment was a little over two months, the bichloride solution being gradually reduced in strength, until at last the 1 to 10,000 was used. At the beginning of the treatment the probe could be passed in to a depth of one and a half inches. At no time did I find any loose pieces of bone.

This case strongly illustrates what a simple alveolar abscess may cause, if neglected, and the importance of thorough, regular, and continuous treatment at the outset. It was also evident that the pus had burrowed along beneath the periosteum, which it had finally penetrated, and involved the submucous tissue at the point of the swelling. At this date, many months after the patient was discharged cured, the parts are still in a normal condition.

*Dr. S. G. Perry.*—I have always distrusted matrices, and yet I have probably used them as much as any one in the profession. I am afraid of them because they prevent access to the cavity, and render the adaptation of the gold to the margins a little uncertain. In the use of plastics, of course, this objection does not apply. I aim to be supplied with a great variety of them, so that I can make a prompt and suitable adaptation to any case. Some time since I showed before this Society some thin steel matrices soldered to a handle and having on one end a lug, which, resting on the adjoining tooth, insured a good fit. I have here a thin steel matrix soldered to a handle, and having on its free end the equivalent of two lugs, so that it can be used between two teeth and will fit each, so that fillings can be put in on each side at the same time, and when the matrix is removed the fillings will both be contoured. I have here also a matrix with the equivalent of the double lug on both ends, but made so as to be adjustable with a screw. Tightening the screw brings the double lugs together until they fit on both sides of the teeth. Loosening the screw enables one to spread the lugs and remove the matrix. Of course, the lugs are filed to fit the contours of the teeth. This is a matrix that will not be often used, but it has its place among the many that can be used to advantage.

Here is a simple matrix which I use when the inner cusp of a bicuspid is broken away, and the two proximal surfaces and the lingual side are to be filled together. It consists of a simple band of steel bent about the tooth and cut away freely on the lingual side of the tooth, to allow access to the two cavities. It confines the plastic filling so that when pressure is applied on one side, the filling does not bulge out on the other. Of course we all know that the quality of an oxyphosphate filling is improved if it is put in a cavity that will hold it while pressure is applied to it, so as to condense it before crystallization occurs. With this style of matrix a filling can be put on the three sides of a tooth with one mixing of the cement. It is too simple to show before this Society, as doubtless many use it in the same way, and yet a man long in practice told me in my office the other day that he had never seen such a matrix used, and that he had been greatly puzzled with the cavities I have described. I show it now, thinking that possibly some one may get a hint from it. I have also here a little strip of rolled gold, about No. 60, which I have used many times as a matrix while filling the front teeth with gutta-percha or oxyphosphate. I suppose with this little piece of gold, used as a matrix, I have filled over fifty teeth. I slip it between the teeth and bind it close to the outside of the tooth I want to fill, and away from the inside of it, so as to allow access to the cavity, which, of course, is generally opened on the inside. I hold it on the outside by the finger while pressing the filling in place.

*Dr. Allan.*—In all cases where the gums are spongy or bleed from any cause, I think a drop of murelene is very good. It stops the bleeding quicker, and does not leave a black deposit that you have to wipe out again. I don't know the nature of the compound, and cannot say anything about it, except that I find it exceedingly useful. It works quicker than any other styptic with which I am acquainted.

*Dr. Francis.*—Does it test acid?

*Dr. Allan.*—I don't know. Now and then patients complain that it gives them pain, but those patients complain of anything I use; so I do not think it is really objectionable. I hope I emphasized the fact that the idea of the electric model I showed you was taken from Dr. Van Woert.

The paper of the evening was then read by Dr. Charles F. Allan, entitled "Eclecticism, the Best Conservative Practice."

(For Dr. Allan's paper, see page 294.)

DISCUSSION.

*Dr. Francis.*—In the course of our lives words sometimes come to our ears that leave an indelible impression upon our minds. At a meeting of the Odontological Society some years ago, where a large number of dentists from different parts of the country were present, the subject of "filling-materials" was largely discussed. There were advocates of soft foil, cohesive foil, sponge gold, amalgam, gutta-percha, etc. Towards the end of the discussion, a gentleman much esteemed in our profession made this remark: "If you thoroughly prepare a cavity for filling and wish to preserve the tooth as long as possible, the best material to use is *good* judgment. Of course, you can deduce the moral.

In regard to Dr. Allan's paper I will simply say, that I listened to it with great interest and fully endorse it throughout.

*Dr. Perry.*—I do not think the paper is one that can be discussed. It commends itself so forcibly to one's common sense, that there is really not much to be said about it, except to commend it. I think, however, that Dr. Allan emphasizes a little too much the importance to be placed upon the fact that a few men do not use amalgam, and are not met with that sweeping condemnation that would be expected. I think the members of the profession are not outspoken on that subject, because they think it is not worth while. I suspect that was the reason that question was not discussed more fully in Chicago, and I think that is why it is not discussed in our societies. There is an undercurrent of common sense in our profession that will not tolerate extremes, and it is useless to try to convince most men that amalgam has no place in dental practice.

One or two points in the paper I wish to speak about. Dr. Allan favors the use of crystal gold. There never was a time in my practice that I have not used it to quite an extent, and I think in later years I have used it more than before. As for cohesive gold, of course I use that, but rarely from the beginning of the cavity to the end. I commence almost all my fillings with the softer forms of gold, using mostly Morgan & Hasting's gold for three-quarters of the filling, finishing off nearly always with crystal gold. I think a good surface can be secured more easily with that, and a better finish. The use of the matrix is very helpful for those soft forms of gold, but is not to be much trusted with the strictly cohesive.

*The President.*—We should like to hear from visitors who are with us to-night, and I will call upon Dr. S. B. Palmer.

*Dr. S. B. Palmer.*—I have very little to say in regard to the paper under discussion, and merely rise to express my gratitude at being with you. I had received a notice, and I did not know that I could pass the evening more agreeably than by coming here. I am very much pleased with the paper. I will speak further later in the evening.

*Dr. Parmley*, of Hartford.—In mentioning the case of putting this extra burden on nervous and anæmic children, it brought one case forcibly to my mind which I have seen in the last six months. The use of the rubber dam, not entirely for the benefit of the patient, but for his comfort as well, struck me forcibly. I have quite often heard patients express their delight that we had discovered something to take the place of the clumsy napkin that was formerly so generally used, and still is by some.

*Dr. Palmer.*—I wish to refer to the electric battery and the cautery point. I use it for drying the root-canals. I would make a suggestion that with the loop at the point, you solder in some of the spring gold, or clasp gold wire, and make it fine so as to enter the nerve-canal. I use that in preference to copper or silver. You require a stiffness to the point similar to steel, and this can be made quite fine. The point of heat is below at the forks, and it only communicates the heat to the little broach that enters to the end of the cavity with a pumping movement. It thus takes up the moisture and immediately evaporates it. You can dry the canal thoroughly as far as the point can be made to enter. The patient will tell when the tooth is warm enough, so there is no danger of overheating. You can bend the broach so as to enter the canals at various angles. In regard to the filling, when the canals are properly dried I take paraffine and harden it with some resinous gum, place a little in the cavity, and on touching it with the heated point it goes home to the root. I first used copper points separately, heating with a dull point, but would have to pull the point back with another instrument. By having two or three different curves, you can change them instantly. One cautery should be blunt, merely the wire loop. To complete the filling have copper or silver points (I prefer pure silver), coat them with paraffine, place in canal, and touch the end with the heated loops. It will go home, and if you ever have occasion to remove it, a mere touch of the excavator will take it out. I generally take eucalyptus or oil of cinnamon and put it at the apex, and then pass in the point and volatilize it as much as possible. One lesson I learned from that, and that was how imperfectly I could dry a root. How much we think we do, when we really do not! My method of doing it was to take a little glass straw, hold it over the Bunsen burner, draw apart, break, and have two artificial root-canals. You will find it a very difficult thing to dry them in. When you first apply the broach they will hiss and boil, but by perseverance you will succeed in drying them. If you use the paraffine, the canal is filled perfectly. I put it in and then fill over with whatever I wish,—chloro-percha or phosphate. I have been most successful with this filling. It is the most accessible in case of trouble.

*Dr. George Allan.*—I have not had this battery long enough to experiment with it in all ways, but I desire to call attention to one thing,—Dr. Palmer having drawn your attention to it,—drying cavities previous to filling. Many of you have heard of obtunding sensibility by using a current of hot air. In two or three cases where I have tried this cautery point,—imperfectly, I acknowledge, —I have found that I obtained very excellent results. Approaching the point to the sensitive dentine in the cavity, I find I can obtund the sensibility to a great extent, and with little pain to my patient. I think that, when it is more thoroughly understood, it will supersede the hot-air current. It is vastly more convenient to use. There is no harm in drawing your attention to it, although I have not experimented very much with it myself. It will dry a cavity with marvellous rapidity and thoroughness. It seems to me it can be used to great advantage by a dentist.

*Dr. Brewster.*—I would like to ask Dr. Palmer, or Dr. Allan, if they have used calium natrium for drying cavities, and if they consider this superior?

*Dr. Palmer.*—I have not.

*Dr Allan.*—Neither have I.

*The President.*—I think the object of that is not to dry cavities.

*Dr. Brewster.*—I understood at Chicago that it was used for getting moisture from the canals.

*Dr. Allan.*—A method I have employed in drying cavities makes use of the same principle adopted by microscopists in preparing mounts. The section or object in water, or some aqueous fluid, is transferred first to alcohol, which abstracts the water, and then placed in the balsam, which in turn is miscible with the balsam. So by the use of absolute alcohol all the water in the root-canal can be abstracted by repeated applications, and then the alcohol, being very volatile, can, by means of the hot-air syringe, be quickly evaporated and the root-canal left in not only a state of complete dryness, but also in an aseptic condition.

*Dr. Perry.*—It should have been said that our President was the first man to call attention to the advantage of using hot air. Where the pulp has been recently devitalized and removed, I think we can fill a tooth with a permanent filling and have no anxiety; but there are a large number of teeth which we can recognize instantly that have been dead for a long time, where there is no certainty of filling them permanently. My method has not been that of Dr. Palmer, but rather the use of a fine piece of gold wire, used with chloro-percha formerly, and later with the chloride of zinc. The little wire can be drawn out and give easy access to the root. I think a great deal can be said about the paper after all. I hope we shall hear from Dr. Bogue and Dr. Howe on the subject. This Society stands for conservatism, and I hope we will hear from them.

*Dr. Bogue.*—There was one remark in the paper that caused me to put down a little note,—it was in regard to the teeth that were tender from wedging, and which became, as he operated upon them, more and more tender, and more and more difficult to work upon. I felt very much like asking Dr. Allan if he does not use a separator and keep them steady, and so avoid the pain. The second thing Dr. Perry has fully answered, which was in regard to crystal gold, which has a surface that can scarcely be excelled. Its adaptability for that purpose is marvellous. I also felt like calling Dr. Allan's attention to the fact that a committee was appointed many years ago to test the relative merits of crystal gold and foil. I happened to be on one of those committees. The result was that many teeth were filled partly with crystal gold and partly with foil. After many years, when one of these teeth came into my possession broken in two, I found the crystal gold and foil equally good, and well adapted to the walls of the cavity, but the crystal gold had taken twice as long as the foil to insert, and the difference in efficiency was not perceptible.

I want to make my thanks to Dr. Palmer, for he has shown to me a new and what seems to me a very good method of filling roots. I have for many years used oxychloride, introduced on a few wisps of cotton wound around a broach, and passed to the end of the canal, leaving the cotton there. I want to ask Dr. Palmer if I understood that in the event of wishing to get at the apex of a root filled with paraffine, on withdrawing the silver wire there is a certain thickness of paraffine between the end of the wire and the apical foramen?

*Dr. Palmer.*—You take a Donaldson broach and hook it out. The end which is in the pulp-cavity is bent at right angle to assist in removal; it is not in the way at all. Where the root is absorbed, if the opening is very large, get your measure and then bend the outer end of wire you use until it goes in even with the end of the root. Take the measure so as not to have the filling pass through. The paraffine will fit in and there will be a good filling without having it thrust through.

*Dr. Parmly.*—Is paraffine a new material for the filling of roots, or has it not been used before? I am very certain that Dr. Tomes many years ago recommended paraffine, but he used it in a different manner.

*Dr. Palmer.*—I do not claim anything new for it. It has been used before.

*Dr. Perry.*—Is there any deterioration?

*Dr. Palmer.*—The batteries used by the telegraph companies I found to be ahead of nearly all the others for the use which they claim. They use paraffine entirely to insulate the wires. The whole wire is passed through paraffine. I think they know whether it deteriorates or not. I do not believe it does.

I prepared those glass tubes, and also took specimens of teeth and dried them and filled them. Take a tooth and put it in water, then cut it open. In experimenting with paraffine, color it with vermilion. You can then see what you are doing; also when you split the tooth open, you will know whether you have it perfect or not.

*The President.*—Inasmuch as Dr. Perry mentioned the subject, I may be permitted to say in reference to the use of heat for obtaining sensitiveness, that while I do not know that I was the first to call attention to it, I did call attention to it a great many years ago in a paper read before the Brooklyn Dental Society. I practised that method for many years. I was led to think of it from the fact which I had observed, that where the rubber dam had been on for some time the teeth became very much less sensitive, and I argued that it came from the drying of the contents of the tubuli, so that sensation could not be conveyed so readily. Acting upon that I improved upon it by using hot air injected into the cavity. I do not use it as much as formerly, because I have the habit latterly of preparing my cavities under a jet of water, which has the double purpose of washing out the bur, keeping it clean and cool, and seems to me to somewhat relieve the disagreeable jarring of the bur. At least, I find comparatively little complaint on the part of patients now of the pain of the operation. I would like to correct Dr. Brewster in regard to the use of kalium-natrium. The use of it was not intended for drying the cavity, but to cleanse the contents of the pulp canal. It does that instantly almost, by changing the putrid contents into soft soap, which can be washed out readily with hot water, and then any means of treating can be adopted that is preferred. I see this method of Dr. Palmer's is an improvement on that of Dr. Evans's root-drier, and I should think it would be very effective.

*Dr. Bogue.*—Following your suggestion of hot air, if the air before being heated is passed through a receptacle containing chloride of lime, which has a strong affinity for water, the air will come into the cavity largely deprived of its moisture; so, being both dry and hot, its work will be more efficient. One must not expose this air to the patient's respiration.

Motion of Dr. Francis for vote of thanks to Dr. Allan unanimously carried.

Adjourned.

John I. Hart, D.D.S.,
*Editor New York Odontological Society.*

